# Supplementary material for: Triplication of the interferon receptor locus contributes to hallmarks of Down syndrome in a mouse model
Source: Nat Genet. 2023 Jun 5;55(6):1034–47. doi: 10.1038/s41588-023-01399-7 (PMC10260402; doi:10.1038/s41588-023-01399-7)
Supplement: Supplementary file 1 — Reporting Summary [file 41588_2023_1399_MOESM1_ESM.pdf]

nature portfolio

Corresponding author(s): Joaquin M. Espinosa and Kelly D. Sullivan

Last updated by author(s): Nov 22, 2022

Reporting Summary

Nature Portfolio wishes to improve the reproducibility of the work that we publish. This form provides structure for consistency and transparency in reporting. For further information on Nature Portfolio policies, see our Editorial Policies and the Editorial Policy Checklist.

Statistics

For all statistical analyses, confirm that the following items are present in the figure legend, table legend, main text, or Methods section.

n/a	Confirmed	
☐	☒	The exact sample size (*n*) for each experimental group/condition, given as a discrete number and unit of measurement
☐	☒	A statement on whether measurements were taken from distinct samples or whether the same sample was measured repeatedly
☐	☒	The statistical test(s) used AND whether they are one- or two-sided<br>*Only common tests should be described solely by name; describe more complex techniques in the Methods section.*
☐	☒	A description of all covariates tested
☐	☒	A description of any assumptions or corrections, such as tests of normality and adjustment for multiple comparisons
☐	☒	A full description of the statistical parameters including central tendency (e.g. means) or other basic estimates (e.g. regression coefficient) AND variation (e.g. standard deviation) or associated estimates of uncertainty (e.g. confidence intervals)
☐	☒	For null hypothesis testing, the test statistic (e.g. *F*, *t*, *r*) with confidence intervals, effect sizes, degrees of freedom and *P* value noted<br>*Give P values as exact values whenever suitable.*
☒	☐	For Bayesian analysis, information on the choice of priors and Markov chain Monte Carlo settings
☒	☐	For hierarchical and complex designs, identification of the appropriate level for tests and full reporting of outcomes
☐	☒	Estimates of effect sizes (e.g. Cohen's *d*, Pearson's *r*), indicating how they were calculated

Our web collection on statistics for biologists contains articles on many of the points above.

Software and code

Policy information about availability of computer code

Data collection	RNA-seq and murine whole genome sequencing data were collected with an Illumina NovaSeq 6000 instrument. Inflammatory marker data were collected with MESO QuickPlex SQ 120 instrument. Sanger sequencing was done using a 3730xl DNA Analyzer (ThermoFisher Scientific). Flow cytometry data were collected with a Cytek® Aurora spectral flow-cytometer and a Accuri C6 flow-cytometer. ELISA data were collected on a Synergy H4 Hybrid Multi-Mode Microplate Reader (BioTek). Heart histology data was collected with a Keyence BZ-X710 All-in-One Fluorescence Microscope. Rotarod data were collected with Rotarod v1.4.1 software (MED Associates, Inc.). Morris water maze data was collected using the video tracking system Ethovision v8.5 (Noldus). Context fear conditioning data were collected with FreezeScan v2.00 (Clever Sys. Inc.). Micro-computed tomography scan data were reconstructed to multiplanar slice data using NRecon v1.7.4.6 (Bruker). Coordinates for craniofacial landmarks were defined with Drishti v2.6.5.
Data analysis	No new software was developed during this study. All data analysis was carried out using existing software as described in the Online Methods for each specific experiment. Software employed in this study includes FASTQC v0.11.5, bcl2fastq v2.20.0.422, FastQ Screen v0.11.0, BBTools v37.99, ea-utils v1.05, HISAT2 v2.1.0, SAMtools v1.5, HTSeq-count v0.6.1, BWA v0.7.15, RSeQC v4.0.0, R 4.0.1, RStudio 2022.02.0, Bioconductor 3.11, the R packages DESeq2 v1.28.1, lmerTest v3.1-2, Hmisc v4.4-0, ggplot2 v3.3.1, fgsea v1.14.0, survival v3.2-7, coxme v2.2-16, emmeans v1.5.1, and broom v0.7.9, CNV-seq v0.2-8, IPA (winter 2022 release), FlowJo v10, LEGENDplex v2021, Rotarod v1.4.1, Ethovision v8.5, FreezeScan v2.00, NRecon v1.7.4.6, Drishti v2.6.5, Photoshop 24.2.0, WinEDMA v2021, GraphPad Prism v8.0.1, Adobe Illustrator v24.1, Microsoft Word v16.70, Microsoft Excel v16.71, and Endnote v20.5.

For manuscripts utilizing custom algorithms or software that are central to the research but not yet described in published literature, software must be made available to editors and reviewers. We strongly encourage code deposition in a community repository (e.g. GitHub). See the Nature Portfolio guidelines for submitting code & software for further information.

Data

Policy information about availability of data

All manuscripts must include a data availability statement. This statement should provide the following information, where applicable:

- Accession codes, unique identifiers, or web links for publicly available datasets
- A description of any restrictions on data availability
- For clinical datasets or third party data, please ensure that the statement adheres to our policy

The minimum dataset required to interpret, verify, and extend the research in this article is made available in the accompanying Source Data files and through public repositories. Mouse WGS data are deposited in the National Center for Biotechnology Information Sequence Read Archive (NCBI-SRA) under BioProject ID PRJNA776534. Human RNA-seq data generated by the Crnic Institute Human Trisome Project are deposited in the Gene Expression Omnibus (GEO) with accession number GSE190125 and also available through the INCLUDE Data Hub (https://portal.includedcc.org/). Human demographics and clinical source metadata is also available through the INCLUDE Data Hub. Murine RNA-seq data was deposited in GEO with the following accession numbers: GSE218883: adult mouse heart tissue; GSE218885: adult mouse brain tissue; GSE218887: embryonic mouse facial mesenchyme tissue; GSE218888: embryonic E12.5 mouse heart tissue; GSE218889: embryonic E18.5 mouse heart tissue; GSE218890: adult mouse mesenteric lymph nodes. All other source data are provided in the Source Data files with this manuscript. Reference datasets employed in this study were mouse reference genome assembly GRCm38 (mm10) with Gencode vM24 basic annotation (https://www.gencodegenes.org/mouse/release_M24.html), and human reference genome assembly GRCh38 (hg38) with Gencode v33 basic annotation. Images have been deposited in the Figshare platform under entries 10.6084/m9.figshare.22317835 (heart histology) and 10.6084/m9.figshare.22317922 (skull morphology). Flow cytometry source data is deposited in Figshare under entry 10.6084/m9.figshare.22320661.

Human research participants

Policy information about studies involving human research participants and Sex and Gender in Research.

Reporting on sex and gender	This study reports data from 400 research participants, 304 with Down syndrome (trisomy 21) (141 female and 163 males) and 96 euploid controls (52 females and 44 males).
Population characteristics	This study reports whole blood transcriptome analysis and measurements of the inflammatory markers CRP and IL6 in plasma samples from research participants enrolled in the Crnic Institute Human Trisome Project (HTP). The cohort employed for this study includes 304 participants with Down syndrome (trisomy 21) and 94 euploid controls. In addition to karyotype (trisomy 21 (T21) versus euploid controls, D21), and sex (male, female) the other key co-variate used in the various analyses was age. The median age and interquartile range values are 27.6 years (14.4 - 38.9) for euploid controls and 23.2 years (15.2 - 32.2) for participants with trisomy 21.
Recruitment	Recruitment into the Crnic Institute Human Trisome Project (HTP) took place at the University of Colorado Anschutz Medical Campus in Aurora, Colorado, USA, as well as multiple conferences in the USA. The study was promoted through scientific presentations at community conferences, IRB-approved flyers, the Human Trisome Project website (www.trisome.org), websites from affiliated organizations (e.g. the Global Down Syndrome Foundation), the DS-Connect registry, and social media. Participants received US$100 compensation per blood draw. Procedures were performed in accordance with IRB guidelines and regulations. Given the focus on recruitment of individuals with Down syndrome, a self-selection bias is unlikely, as recruitment is based on karyotype, which is confirmed from review of electronic health records.
Ethics oversight	Research participants were enrolled to the Crnic Institute Human Trisome Project Biobank (HTP) under a study protocol approved by the Colorado Multiple Institutional Review Board (IRB; COMIRB #15-2170). Procedures were performed in accordance with COMIRB guidelines and regulations. Written informed consent was obtained from participants who were cognitively able or by guardians of each participant. The study was conducted in accordance with the Declaration of Helsinki.

Note that full information on the approval of the study protocol must also be provided in the manuscript.

Field-specific reporting

Please select the one below that is the best fit for your research. If you are not sure, read the appropriate sections before making your selection.

☒ Life sciences ☐ Behavioural & social sciences ☐ Ecological, evolutionary & environmental sciences

For a reference copy of the document with all sections, see nature.com/documents/nr-reporting-summary-flat.pdf

Life sciences study design

All studies must disclose on these points even when the disclosure is negative.

Sample size	Sample size was determined a priori based on effect sizes of previous studies or by post hoc analyses to ensure >80% power was achieved to reduce type II error (e.g., heart histology, developmental milestone achievement, and cognition in adult mice). Key references we employed to determine the sample size of the human research studies are:<br>Sullivan, K. D. et al. Trisomy 21 consistently activates the interferon response. Elife 5 (2016). https://doi.org:10.7554/eLife.16220<br>Powers, R. K. et al. Trisomy 21 activates the kynurenine pathway via increased dosage of interferon receptors. Nat Commun 10, 4766 (2019).

https://doi.org:10.1038/s41467-019-12739-9
Waugh, K. A. et al. Mass Cytometry Reveals Global Immune Remodeling with Multi-lineage Hypersensitivity to Type I Interferon in Down Syndrome. Cell reports 29, 1893-1908 e1894 (2019). https://doi.org:10.1016/j.celrep.2019.10.038
Araya, P. et al. Trisomy 21 dysregulates T cell lineages toward an autoimmunity-prone state associated with interferon hyperactivity. Proc Natl Acad Sci U S A 116, 24231-24241 (2019). https://doi.org:10.1073/pnas.1908129116
Sullivan, K. D. et al. Trisomy 21 causes changes in the circulating proteome indicative of chronic autoinflammation. Scientific reports 7, 14818 (2017). https://doi.org:10.1038/s41598-017-13858-3

Data exclusions

In high throughout measurements of CRP and IL6, extreme technical outliers were classified per-karyotype and per-analyte as measurements more than three times the interquartile range below or above the first and third quartiles, respectively, and excluded from further analysis. In measurements of cytokines by flow cytometry in murine samples, values are only shown for cytokines detected above background. All other data are included for all experiments.

Replication

All results presented are derived from multiple independent biological replicates, and in the case of mouse studies, also across multiple independent experiments. The numbers of human research participants, animals, and replicates is indicated for each experiment in the corresponding in the Methods and figure legends.

Randomization

For developmental milestones in neonates and cognitive assessment of adults, animals were assessed in a pseudorandom order. For all other murine experiments, animals were evaluated by litter as they became available, and randomized by taking key covariates into consideration including genotype, age, and sex.

Blinding

The investigators who sectioned embryos and performed histology analysis were blinded to embryo genotype to detect heart malformations. The investigators who assessed developmental milestones and cognitive phenotypes were blind to genotype. Craniofacial morphology was assessed by two investigators blinded to genotype. For all other experiments, data generation was done blinded to karyotype (e.g. for human studies of transcriptome and inflammatory markers) or mouse genotype (e.g., transcriptome analyses, flow cytometry) and the key variable was not unmasked until data analysis to define the effect of the variable.

Reporting for specific materials, systems and methods

We require information from authors about some types of materials, experimental systems and methods used in many studies. Here, indicate whether each material, system or method listed is relevant to your study. If you are not sure if a list item applies to your research, read the appropriate section before selecting a response.

Materials & experimental systems

n/a	Involved in the study	
☐	☒	Antibodies
☒	☐	Eukaryotic cell lines
☒	☐	Palaeontology and archaeology
☐	☒	Animals and other organisms
☐	☒	Clinical data
☒	☐	Dual use research of concern

Methods

n/a	Involved in the study	
☒	☐	ChIP-seq
☐	☒	Flow cytometry
☒	☐	MRI-based neuroimaging

Antibodies

Antibodies used

Antibodies for flow cytometry:

Mouse CD4-BV711 (BioLegend #100550, clone RM4-5, RRID:AB_2562607, dilution 1:600); Mouse IA/IE-BV650 (BioLegend #107641, clone M5/114.15.2, RRID:AB_2565975, dilution 1:4800); Mouse Ly6C-BV605 (BioLegend #128036, clone HK1.4, RRID:AB_2562353, dilution 1:600); Mouse CD8-BV510, (BioLegend #100751, clone 53-6.7, RRID:AB_2561389, dilution 1:100); Mouse CD115-BV421, (BioLegend #135513, AFS98, RRID:AB_2562667, dilution 1:100); Mouse CD45-Pacific Blue (BioLegend #109820, clone 104, RRID:AB_492872, dilution 1:400); Mouse SiglecF-BB515 (BD Biosciences #564514, clone E50-2440, RRID:AB_2738833, dilution 1:100); Mouse CD11b-AF532 (Invitrogen #58-0112-82, clone M1/70, RRID:AB_2811905, dilution,1:800); Mouse NK1.1-BB700 (BD Biosciences #566502, clone PK136; RRID:AB_2744491, dilution 1:100); Mouse CD3-PE/Cy7 (BioLegend #100220, clone 17A2, RRID:AB_1732057, dilution 1:100); Mouse Ly6G-BV605 (BioLegend #127623, clone 1A8, RRID:AB_10645331, dilution 1:600); Mouse B220-AF700 (Invitrogen #56-0452-82, RA3-6B2, RRID:AB_891458, dilution 1:400); Mouse IFNAR1-PE (BioLegend #127312, MAR1-5A3, RRID:AB_2248800, dilution 1:50); Mouse IgG1k- PE (Invitrogen #MA1-10407, clone MOPC-21, RRID:AB_2536775, dilution 1:50); Mouse IFNGR2-PE (BioLegend #113603, clone MOB-47, RRID:AB_313560, dilution 1:10); Armenian hamster IgG-PE (BioLegend #400907, clone HTK888, RRID:AB_326593, dilution 1:10); Mouse IL10RB-PE, (Miltenyi #130-114-497, clone REA856, RRID:AB_2726668, dilution 1:10); REA control human IgG1-PE (Miltenyi #130-113-462, clone REA293, RRID:AB_2751113, dilution 1:10): phospho-STAT1 (Tyr701)-PE, (BD Biosciences #562069, clone 4a, RRID:AB_399855, dilution 1:10)

Antibodies for Western blots:

STAT1 (Cell Signaling Technology #9172, RRID:AB_2198300, dilution 1:1000); phospho-STAT1 (Tyr701) (Cell Signaling Technology #9167S, clone 58C6, RRID:AB_561284, dilution 1:1000); GAPDH (Thermo #AM4300, clone 6C5, RRID:AB_2536381, dilution 1:5000); Goat anti-mouse (Southern Biotech #1031-05, RRID:AB_2794307, dilution 1:2000); Goat anti-rabbit (Thermo #65-6120,

RRID:AB_2533967,dilution 1:2000)

Validation

All flow cytometry antibodies were titrated by authors with appropriate positive and negative internal cell population controls as well as isotype and fluorescent minus one (FMO) negative controls when geometric mean fluorescent intensities are reported. Additional validation information from the manufacturers is as follows:

For mouse CD4 - BioLegend Cat #100550, Clone RM4-5, RRID:AB_2562607; Mouse IA/IE - BioLegend Cat #107641, Clone M5/114.15.2, RRID:AB_2565975; Mouse Ly6C - BioLegend Cat #128036, Clone HK1.4, RRID:AB_2562353; Mouse CD8 - BioLegend Cat #10751, Clone 53-6.7, RRID:AB_2561389; Mouse CD115 - BioLegend Cat #135513, Clone AFS98, RRID:AB_2562667; Mouse CD45 - BioLegend Cat #109820, Clone 104, RRID:AB_492872; Mouse CD3 - BioLegend Cat #100220, Clone 17A2, RRID:AB_1732057; Mouse Ly6G - BioLegend Cat #127623, Clone 1A8, RRID:AB_10645331; Mouse IFNAR1 - BioLegend Cat #127312, Clone MAR1-5A3, RRID:AB_2248800; Mouse IFNGR2 - BioLegend Cat #113603, Clone OB-47, RRID:AB_313560; and armenian hamster IgG - BioLegend Cat #400907, Clone HTK888, each lot of these antibodies are quality control tested by the manufacturer by immunofluorescent staining with flow cytometry analysis.

For mouse SiglecF, BD Biosciences Cat #564514, Clone E50-2440, RRID:AB_2738833; Mouse NK1.1, BD Biosciences Cat #566502, Clone PK136, RRID:AB_2744491; phospho-STAT1 (Tyr701), and BD Biosciences Cat #562069, Clone 4a, RRID:AB_399855, these antibodies were validated by immunofluorescent staining with flow cytometry analysis versus isotype controls.

For mouse CD11b, Invitrogen Cat #58-0112-82, Clone M1/70, RRID:AB_2811905; Mouse B220, Invitrogen Cat #56-04522-82, Clone RA3-6B2, RRID:AB_891458; Mouse IgG1k, and Invitrogen Cat #MA1-10407, Clone MOPC-21, RRID:AB_2536775, these antibodies were validated by immunofluorescent staining with flow cytometry analysis versus isotype controls.

For, mouse IL10Rb, Miltenyi Cat #130-114-497, Clone REA856, RRID:AB_2726668; and REA control human IgG1, Miltenyi Cat #130-113-462, Clone REA293, RRID:AB_2751113, these antibodies were validated by immunofluorescent staining with flow cytometry analysis versus isotype controls.

All antibodies used for Western blots were validated by manufacturers as follows: STAT1, Cell Signaling Technology Cat# 9172, RRID:AB_2198300, was validated using STAT1 knockout cell lines; phospho-STAT1 (Tyr701), Cell Signaling Technology Cat# 9167, Clone, 58C6, RRID:AB_2198300, was validated using HeLa cells treated with IFN; GAPDH, Thermo Cat# AM4300, Clone 6C5, RRID:AB_2536381, was validated with Lysates from human, mouse, and rat lysates.

References supporting the validation statements: Harsha Krovi S, et al. 2020. Nat Commun. 4.790277778; Ferrere G, et al. 2021. JCI Insight; Miranda K, et al. 2022. iScience. 25:104994; Luo J, et al. 2022. J Nanobiotechnology. 20:228; Schloss MJ, et al. 2022. Nat Immunol. 23:605; Sandu I, et al. 2020. Nat Commun. 11:4454; Qi Z, et al. 2022. Nat Commun. 13:182; Zaman R, et al. 2021. Immunity; Banks DA, et al. 2019. J Immunol. 202:2348; Su Y, et al. 2022. J Hematol Oncol; Zenke S, et al. 2022. Nat Commun; Angata T, et al. 2001. J Biol Chem. 276(48); Arase N, et al. 1997. J Exp Med. 186(12); Perez OD, et al. 2005. Curr Protoc Cytom.; Verheijden S, et al. 2015. Glia. 63(9); Cheng N, et al. 2008. PLoS One. 3(4); Kang S, et al. 2016. PLoS One. 11(9); Spencer S.D. et al., 1998. J Exp Med. 187(4); Mehl JL, et al. 2022. iScience; Wu W-Y, et al. 2022. Molecules; Awad PN, et al. 2018. Cereb Cortex. 28(11).

Animals and other research organisms

Policy information about studies involving animals; ARRIVE guidelines recommended for reporting animal research, and Sex and Gender in Research

Laboratory animals

The Dp(16Lipi-Zbtb21)1Yey/J mouse model of Down syndrome was originally purchased from The Jackson Laboratory (Cat# JAX:013530, RRID:IMSR_JAX013530) as well as gifted from Dr. Diana Bianchi's lab (National Institutes of Health, NIH) than intermixed and maintained on the C57BL/6J background (The Jackson Laboratory). C57BL/6NTac zygotes (Taconic) were used to generate the mutant mice. Experiments were done animals harvested at embryonic day (E)10.5 (facial mesenchyme), E.12.5 (embryonic heart transcriptome), E15.5 (embryonic heart histology), E.18.5 (embryonic heart transcriptome), day 3-21 post-birth (developmental milestone and size/growth measurements), and ~4-6 months of age (cognitive testing, poly(I:C) treatment, transcriptome studies for mesenteric lymph nodes, adult heart), 5-9 months of age (flow cytometry), 3-9 months of age (ELISA), ~6-9 months of age (brain transcriptome), and 7-10 weeks of age (craniofacial morphology). Mice were housed separately by sex in groups of 1-5 mice/cage under a 14:10 light:dark cycle with controlled temperature and 35% humidity had ad libitum access to food (6% fat diet) and water.

Wild animals

None

Reporting on sex

We carefully report on sex and how data were analysed to analyze and/or account for potential differences by sex.

Field-collected samples

None

Ethics oversight

All animal experiments were approved by the Institutional Animal Care and Use Committee (IACUC) at the University of Colorado Anschutz Medical Campus, under Protocol #00111 and performed in accordance with the NIH guidelines for the care and use of animals in research.

Note that full information on the approval of the study protocol must also be provided in the manuscript.

Clinical data

Policy information about clinical studies

All manuscripts should comply with the ICMJE guidelines for publication of clinical research and a completed CONSORT checklist must be included with all submissions.

Clinical trial registration	Not applicable.
Study protocol	Research participants were enrolled to the Crnic Institute Human Trisome Project Biobank (HTP) under a study protocol approved by the Colorado Multiple Institutional Review Board (IRB; COMIRB #15-2170).
Data collection	Samples were collected through the Crnic Institute Human Trisome Project (HTP) at either the University of Colorado Anschutz Medical Campus in Aurora, Colorado, USA, or at multiple conferences in the USA.
Outcomes	The main goals of the clinical study were to determine baseline changes in circulating inflammatory markers of people with Down syndrome (DS, trisomy 21; T21) 1) in comparison to typical (D21) controls and 2) among individuals with T21. Please see each analysis section for details on RNA-seq and cytokine protein analyses.

Flow Cytometry

Plots

Confirm that:

☒ The axis labels state the marker and fluorochrome used (e.g. CD4-FITC).

☒ The axis scales are clearly visible. Include numbers along axes only for bottom left plot of group (a 'group' is an analysis of identical markers).

☒ All plots are contour plots with outliers or pseudocolor plots.

☒ A numerical value for number of cells or percentage (with statistics) is provided.

Methodology

Sample preparation	Peripheral blood was collected from the submandibular vein of mice 17-25 weeks of age into tubes of lithium heparin (Sarstedt, Cat#41.1393.105) then stained as previously described with minor alterations.
Instrument	Cells were analyzed using a five laser Cytek® Aurora spectral flow-cytometer.
Software	Flow cytometry data was similarly analyzed with FlowJo Software (Becton, Dickinson & Company).
Cell population abundance	Cells were not sorted. Only the geometric mean fluorescent intensity is compared between the immune cell populations enriched by flow-cytometry according to Extended Data Fig. 3d instead of cell population abundance.
Gating strategy	Please see Extended Data Fig. 3d for the gating strategy. Briefly, white blood cells were gated by CD45+; Eosinophils and Neutrophils by scatter, Ly6G and SiglecF; B cells by B220; T cell subsets by CD3, CD4, and CD8; NK cells by NK1.1; Monocytes by CD115 and CD11b; then monocyte subsets by Ly6C.

☒ Tick this box to confirm that a figure exemplifying the gating strategy is provided in the Supplementary Information.
