# Supplementary figures and images for: Triplication of the interferon receptor locus contributes to hallmarks of Down syndrome in a mouse model
Source: Nat Genet. 2023 Jun 5;55(6):1034–47. doi: 10.1038/s41588-023-01399-7 (PMC10260402; doi:10.1038/s41588-023-01399-7)

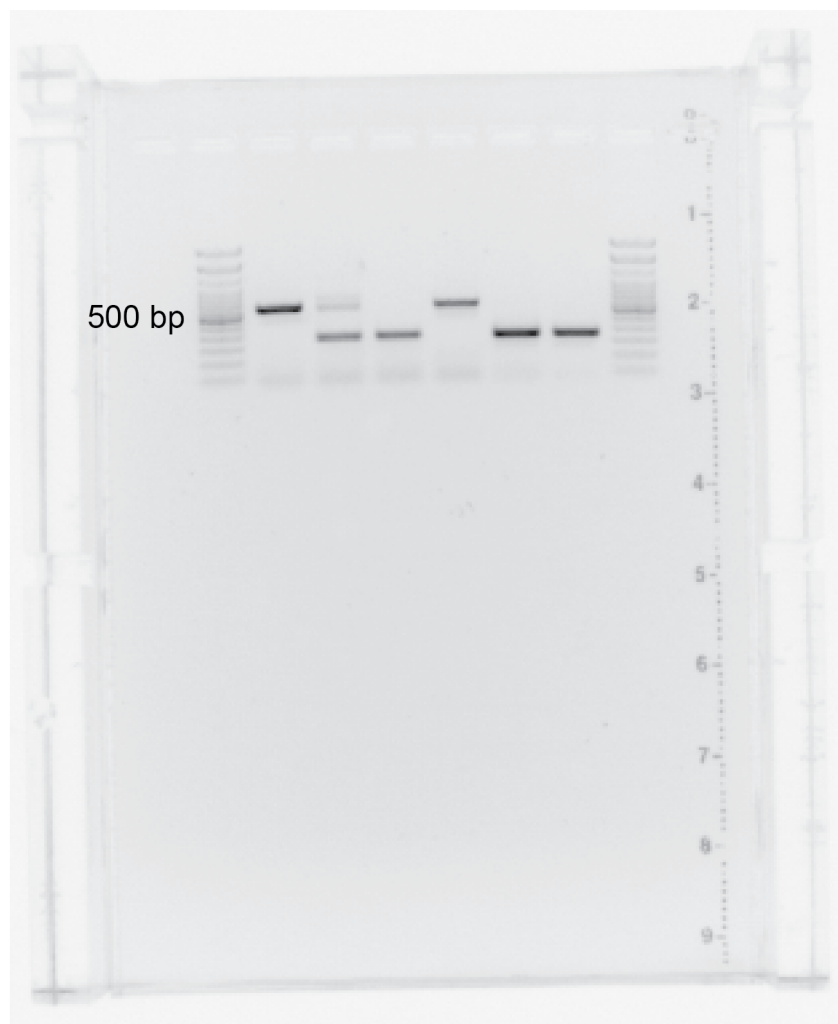

Supplement: Source Data Extended Data Fig. 2 — Unprocessed gel image. [file 41588_2023_1399_MOESM18_ESM.pdf]

# Gapdh

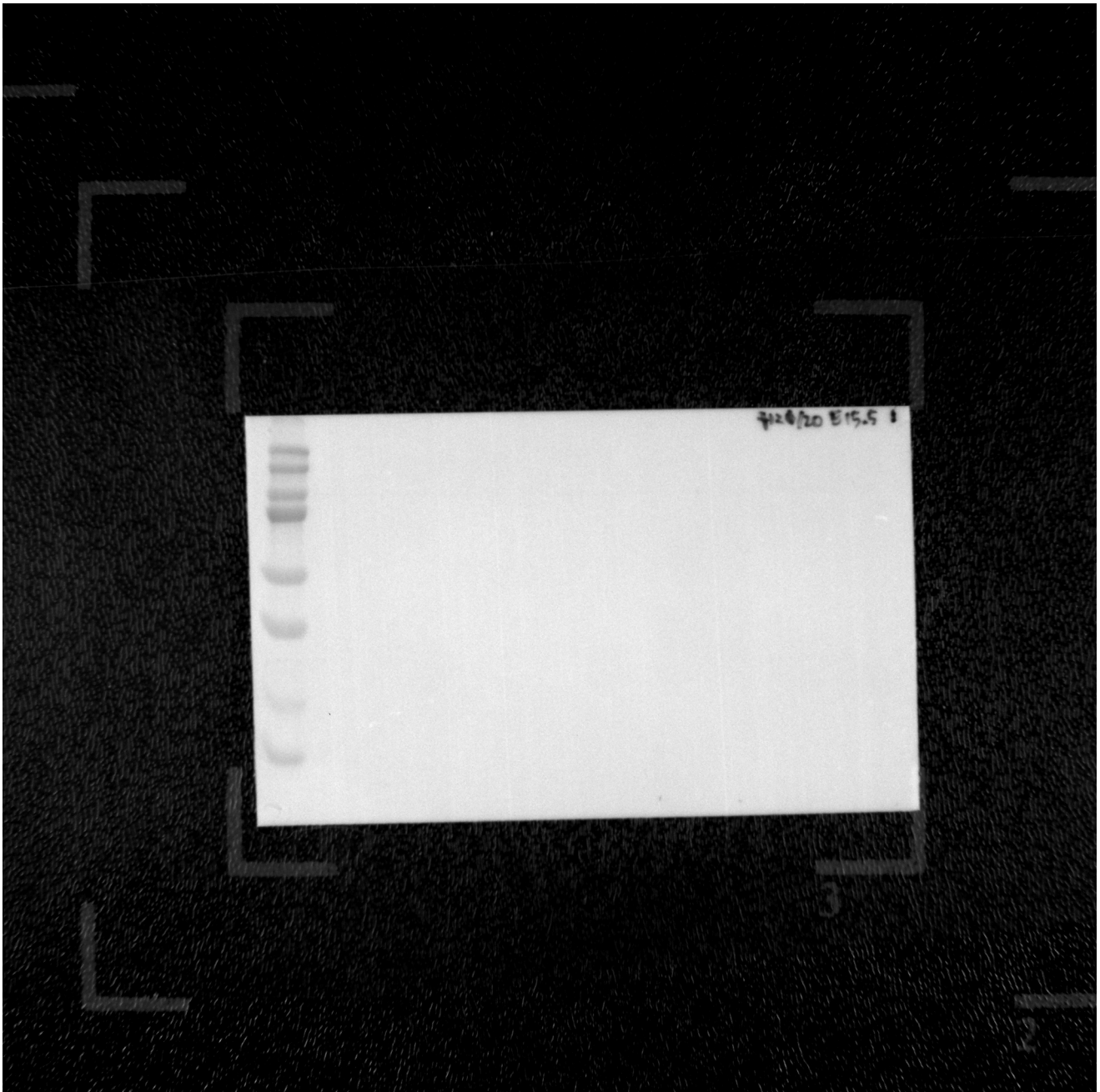

Gapdh

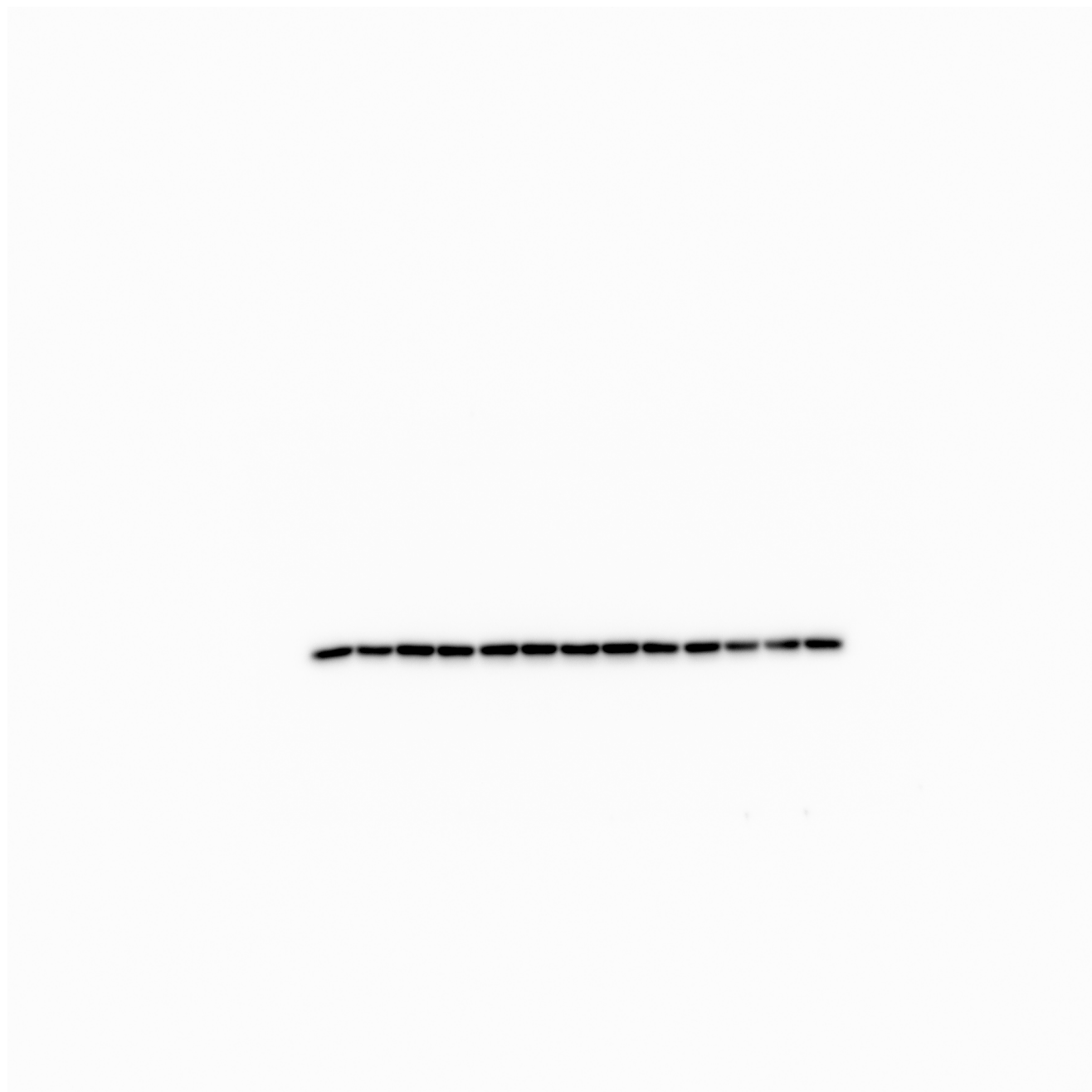

# Gapdh

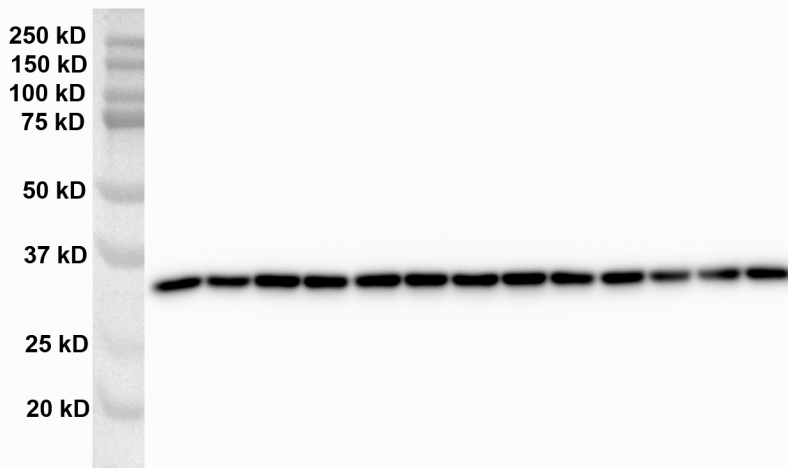

pStat1

15613 02/02/14

1111 1 1 1 1

pStat1

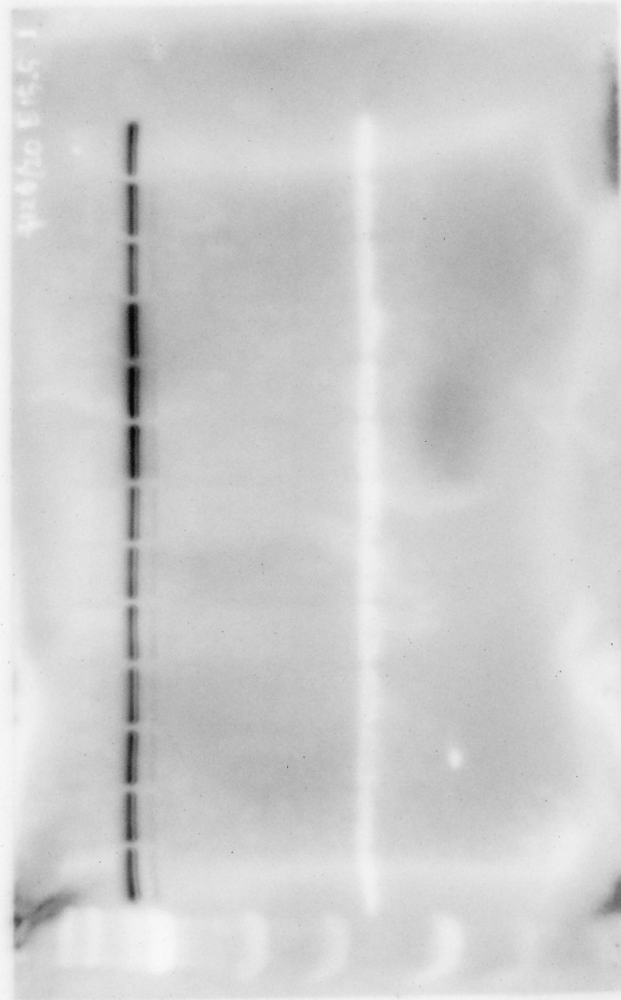

pStat1

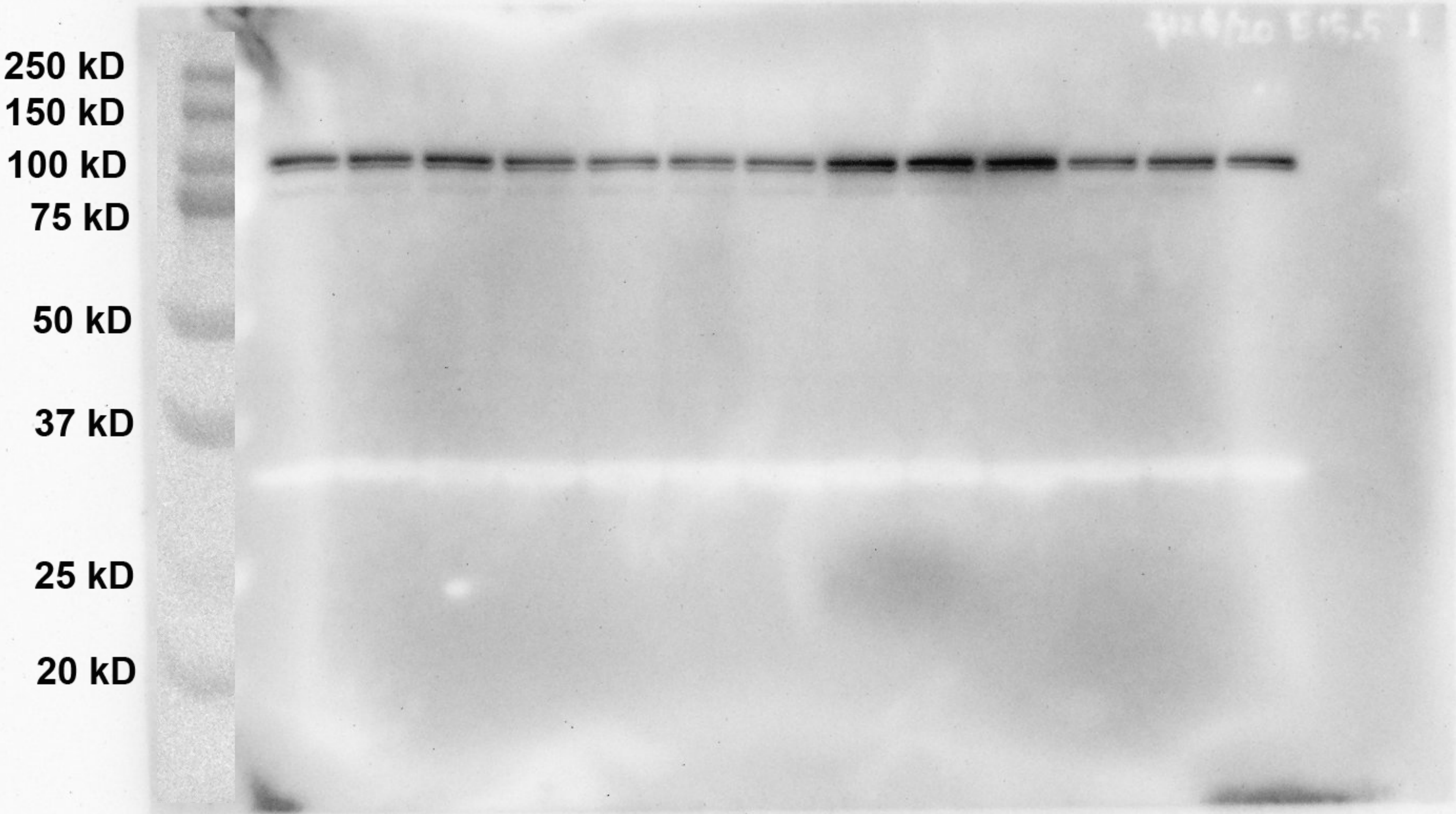

Stat1

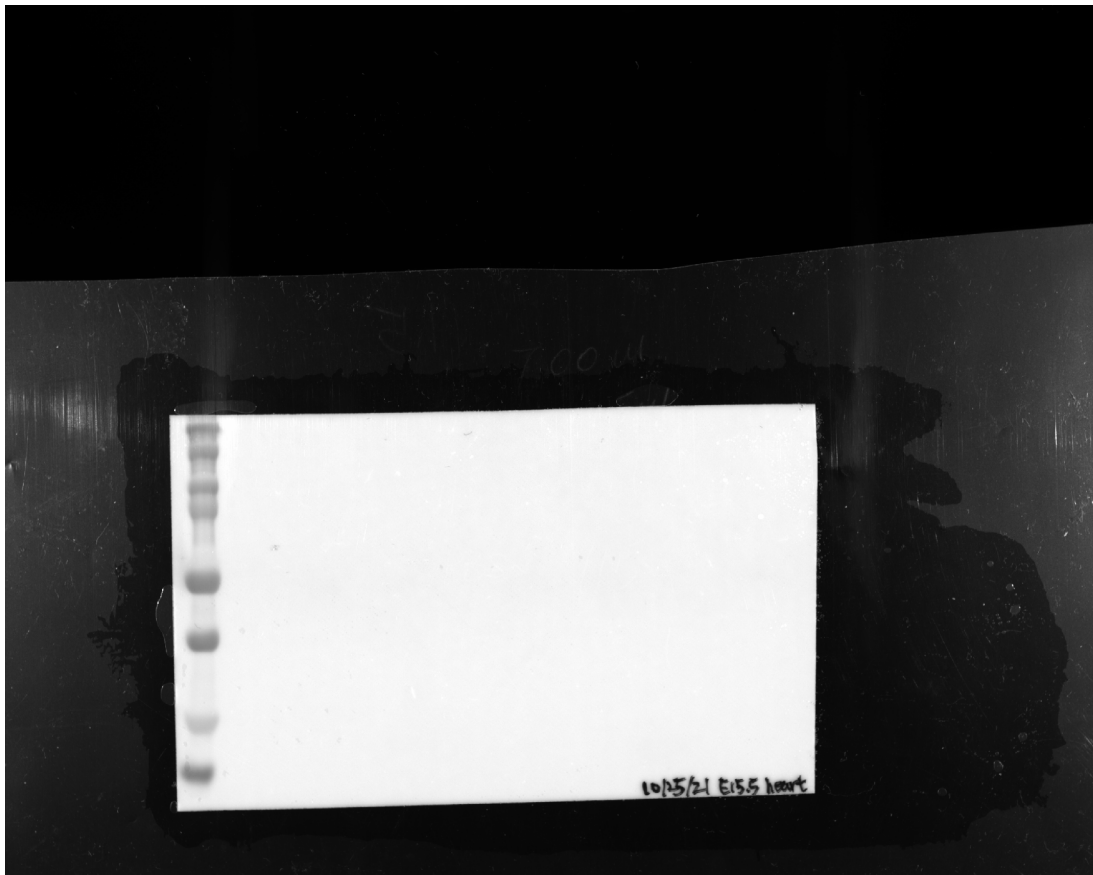

Stat1

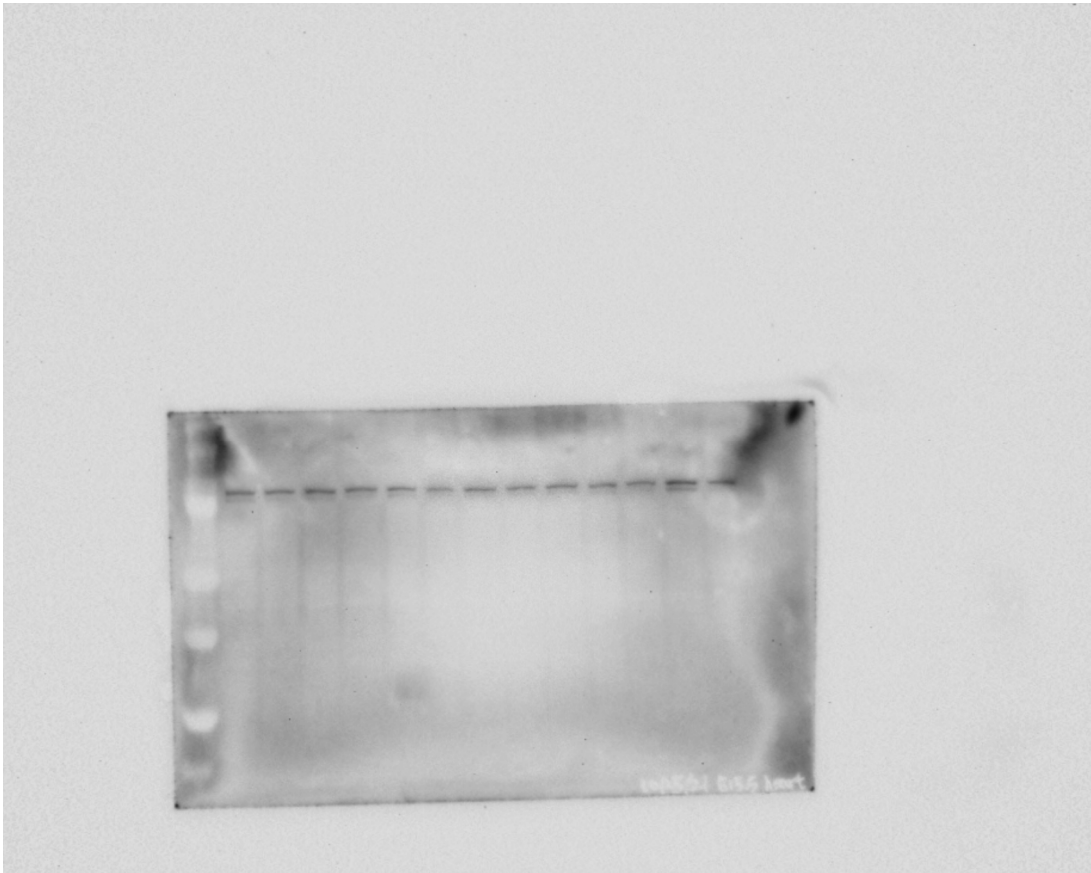

# Stat1

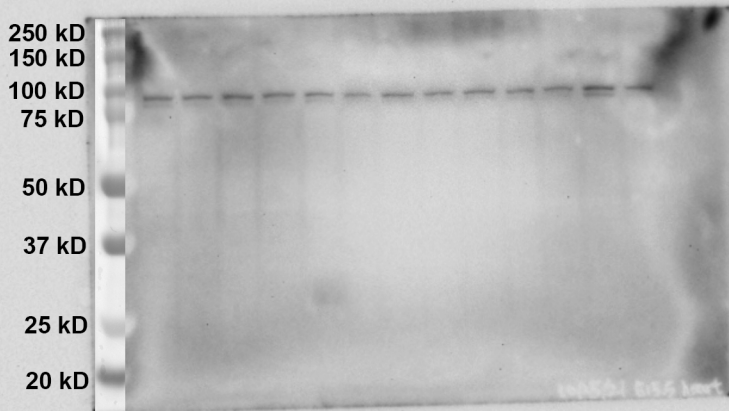

Supplement: Source Data Extended Data Fig. 4 — Unprocessed western blots. [file 41588_2023_1399_MOESM21_ESM.pdf]
